# Diagnostic ability of confocal near-infrared reflectance fundus imaging to detect retrograde microcystic maculopathy from chiasm compression. A comparative study with OCT findings

**DOI:** 10.1371/journal.pone.0253323

**Published:** 2021-06-24

**Authors:** Mário L. R. Monteiro, Rafael M. Sousa, Rafael B. Araújo, Daniel Ferraz, Mohammad A. Sadiq, Leandro C. Zacharias, Rony C. Preti, Leonardo P. Cunha, Quan D. Nguyen

**Affiliations:** 1 Division of Ophthalmology and the Laboratory for Investigation in Ophthalmology (LIM-33), Faculdade de Medicina FMUSP, Universidade de São Paulo, São Paulo, Brazil; 2 NIHR Biomedical Research Centre for Ophthalmology, Moorfields Eye Hospital NHS Foundation Trust and UCL Institute of Ophthalmology, London, United Kingdom; 3 Department of Ophthalmology, University of Louisville, Louisville, Kentucky, United States of America; 4 Department of Ophthalmology, Federal University of Juiz de Fora Medical School, Juiz de Fora, Minas Gerais, Brazil; 5 Byers Eye Institute, Stanford University, Palo Alto, California, United States of America; Massachusetts Eye & Ear Infirmary, Harvard Medical School, UNITED STATES

## Abstract

**Purpose:**

To evaluate the ability of confocal near-infrared reflectance (NIR) to diagnose retrograde microcystic maculopathy (RMM) in eyes with temporal visual field (VF) loss and optic atrophy from chiasmal compression. To compare NIR findings with optical coherence tomography (OCT) findings in the same group of patients.

**Methods:**

Thirty-four eyes (26 patients) with temporal VF loss from chiasmal compression and 41 healthy eyes (22 controls) underwent NIR fundus photography, and macular OCT scanning. VF loss was estimated and retinal layers thickness were measured on OCT. Two examiners blinded to the diagnosis randomly examined NIR images for the presence of hyporeflective abnormality (HA) and OCT scans for the presence of microcystic macular abnormalities (MMA). The total average and hemi-macular HA area and number of microcysts were determined. The groups were compared and the level of agreement was estimated.

**Results:**

The OCT-measured macular retinal nerve fiber and ganglion cell layers were thinner and the inner nuclear layer was thicker in patients compared to controls. HA and MMA were detected in 22 and 12 patient eyes, respectively, and in 0 controls (*p*<0.001, both comparisons). HA was significantly more frequent than MMA in patients with optic atrophy, and agreement between HA and MMA (both total and hemi-macular) was fair (kappa range: 0.24–0.29). The mean HA area was significantly greater in the nasal than temporal hemi-macula. A re-analysis of the 14 eyes with discrepant findings allowed to confirm RMM in 20 eyes (20/34) indicating that OCT detected RMM in 12 and missed it in 8 eyes. On the other hand, NIR correctly detected 18 out of 20 eyes, overcalled 4 and missed 2.

**Conclusions:**

RMM is a frequent finding in eyes with severe VF loss from long-standing chiasmal compression. NIR photography appears to be more sensitive than OCT for detecting RMM and may be useful as screening method for its presence.

## Introduction

Retrograde microcystic maculopathy (RMM) is a recently described macular abnormality occurring in many anterior visual pathway diseases including demyelinating, hereditary, nutritional and compressive optic neuropathies and glaucoma [1–3]. Although fundoscopy and color fundus photography do not reveal abnormalities in the macula in such conditions, advances in optical coherence tomography (OCT) have made it possible to detect RMM by the visualization of microcysts in the inner nuclear layer (INL) of the macula, an OCT abnormality that has also been termed microcystic macular abnormality (MMA) [2,4–7]. Initially thought to indicate retinal inflammation, RMM is now considered a degenerative retinal condition [1,2,8] in response to retrograde trans-synaptic ganglion cell degeneration in optic neuropathies [9,10]. It is therefore a retinal sign of optic neuropathy caused by different etiologies [2]. However, while RMM is secondary to anterior optic pathway diseases, retinal microcysts may be observed in several other retinal diseases, especially edematous maculopathies.

Macular disease assessment methods include multimodal imaging using not only OCT, color fundus photography and fluorescein angiography, but also technologies based on monochromatic laser-produced wavelengths of the electromagnetic spectrum that improve disease detection by focusing on different depths in the retina [11]. Confocal scanning laser near-infrared reflectance (NIR) fundus imaging provides good images of deeper retinal structures [11–13] and is capable of detecting hyporeflective abnormalities (HA) in patients with OCT-detected MMA [2,3,7,14]. Kaufhold et al. [7] retrospectively reviewed the OCT findings of 283 patients with demyelinating diseases and found MMA in 22 eyes of 15 patients (5.3%). In all 22 eyes, HA was present on NIR when evaluated by an independent examiner blinded to the OCT findings. No study, however, has prospectively investigated the diagnostic ability of NIR to detect RMM in compressive optic neuropathies or compared its sensitivity to that of OCT for detecting retrograde macular degeneration in such patients. Defined based on OCT findings, MMA frequently requires high-resolution linear B-scans to be detected and may not be easy to visualize on lower-resolution scans obtained with volumetric macular OCT scanning protocols which are most frequently used for quantifying retinal ganglion cell layer in optic neuropathies. Also, OCT detection of MMA usually requires a careful and time-consuming search of individual high-resolution line scans. On the other hand, since NIR is a fast procedure associated with minimal patient discomfort, it is relevant to evaluate its usefulness as a screening method for RMM. Moreover, a better understanding of RMM-related findings on NIR can help distinguish RMM from other macular diseases.

Patients with permanent temporal hemianopia from chiasmal compression display a rather specific pattern of retinal degeneration in which neural elements from the nasal hemi-retina (corresponding to the temporal field) are significantly more damaged than their temporal counterparts [15–17]. In such eyes there is preferential loss of axons originating in the nasal hemi-retina, which cross in the chiasm, with relative sparing of the axons arising in the temporal hemi-retina, a pattern known as band atrophy (BA) of the optic nerve [18]. Since the retinal nerve fibers originating in the nasal hemi-retina and their corresponding ganglion cells are preferentially damaged by tumor compression of the crossed chiasmal fibers, the patterns observed in the two hemi-retinas are distinct, providing an important framework for comparisons [15,18].

The purpose of the present study was to conduct a masked investigation of HA on NIR fundus photography and MMA on OCT in the macula of patients with temporal VF defects and BA of the optic nerve from compressive chiasmal lesions and compare the outcome of the two methods. We also assessed the agreement between measurements of the whole macula and the temporal and nasal hemi-maculae, and evaluated possible correlations with VF loss and OCT-measured inner retinal layer abnormalities secondary to retinal neural loss from chiasmal compression.

## Methods

The study was approved by the Institutional Review Board Ethics Committee at our institution (*CAPPesq*, Approval Number: 23744813.0.0000.0068). It followed the principles of the Declaration of Helsinki and written informed consent was obtained from all participants. The study was observational, prospective cross-sectional and included 26 patients with a history of treatment of suprasellar lesions and complete or partial temporal VF defects on standard automated perimetry, stable for at least one year prior to study entry. Temporal VF defect was defined as the presence of at least two non-edge contiguous test points in the temporal field, with a pattern deviation (PD plot) of one point with P < 0.5% and one point with P < 2%. Patients were scanned using magnetic resonance imaging (MRI) to confirm the diagnosis of tumor compressing the optic chiasm and to document effective optic pathway decompression after treatment. Additional inclusion criteria included: best-corrected visual acuity of 20/30 or better in the study eye, spherical refraction of ±5 diopters for the most ametropic meridian, a normal nasal hemifield on standard automated perimetry [19], intraocular pressure <22 mmHg and reliable performance on VF testing. Patients with a history of intraocular diseases or with clinical signs of maculopathies, glaucoma or other optic disc anomalies were excluded.

The control group consisted of 22 normal healthy volunteers recruited from among the hospital staff. Control subjects had normal ophthalmic findings and normal VF. A normal VF was defined as a pattern standard deviation (PSD) within the 95% confidence limits and a Glaucoma Hemifield Test result within normal limits. Both eyes of each healthy subject were included for analysis, with the exception of one eye excluded because of sectorial lens opacity. Perimetry was performed on the same day as the OCT testing.

VF testing was undertaken using the 24–2 SITA-Standard strategy (Humphrey Field Analyzer, Carl-Zeiss Meditec, Dublin, CA, U.S.A.) and a Goldmann size III stimulus. Reliability criteria were false positives, false negatives or fixation losses smaller than 30%. The severity of VF defects in patients with BA was assessed by evaluating mean VF sensitivity loss for 50 of the 24–2 standard test locations, excluding the 2 points immediately above and below the blind spot, as global mean deviation (MD) based on the total deviation plot (in dB) of the SAP. VF loss was also calculated as mean deviation and for each hemifield (nasal = 26 test points, temporal = 24 test points), labeled as nasal mean deviation (NMD) and temporal mean deviation (TMD).

### Optical coherence tomography

The subjects were submitted to spectral-domain OCT scanning of the macular and optic disc area after dilating the pupil, using commercially available equipment (Spectralis OCT, Heidelberg Engineering, software v. 5.2). All scans were acquired by an experienced examiner and were reviewed with respect to their subjective and objective quality. The scanning protocol involved the acquisition of: i) a cube scan made in the dense protocol with high-definition OCT images of the macula and optic disc area in a raster pattern covering 20° x 20°, and ii) 49 line scans, each with 16 frames and separated by 120 micrometers, 40,000 axial scans per second at an axial resolution of ~5 micrometers (1,059 A-scans) acquired using the automated eye alignment-tracking software (TruTrack, Heidelberg Engineering, Heidelberg, Germany). To be acceptable, OCT fundus images were required to have an objective quality grade better than 20 in the acquisition mode and to have sufficient subjective quality.

Heidelberg Spectralis data was exported to a personal computer and analyzed with Orion OCT image analysis (Orion^™^, Voxeleron LLC, Pleasanton, CA, USA). This fully-automated software uses deep-learning-based approach for OCT segmentation and is able to measure volumes of retinal layers with distinct boundaries, segmenting up to eight retinal layers in OCT images [20,21]. In our study, the software segmented each of the 49 horizontal scans defining the different layers of the retina. The following retinal layers were measured: i) the macular retinal nerve fiber layer (RNFL), ii) the combination of the ganglion cell layer and the inner plexiform layer (GCL+), and iii) the inner nuclear layer (between the inner plexiform layer and the outer plexiform layer). All scans were evaluated for correct segmentation according to the OSCAR-1B criteria [22]. Thickness values were averaged globally and for each hemi-macula (temporal and nasal).

OCT data from patients and controls were evaluated for the presence of MMA, defined as cystic, lacunar areas of hyporeflectivity with clear boundaries within the INL present on two or more consecutive B-scans [4]. Two examiners, blinded to clinical status of the eye, assessed each one of the 49 scans of each of the 75 study eyes for the presence of MMA and, if present, counted the number of microcysts and recorded their location in the nasal or temporal hemi-macula. When the examiners disagreed regarding the presence or absence of MMA, the case was reviewed by a third examiner equally blinded to the clinical status of the eye. Labeling an eye as normal or abnormal required the agreement of two examiners. The number of microcysts observed by the examiners was averaged.

### Confocal near-infrared reflectance fundus imaging

**Fig 1:**
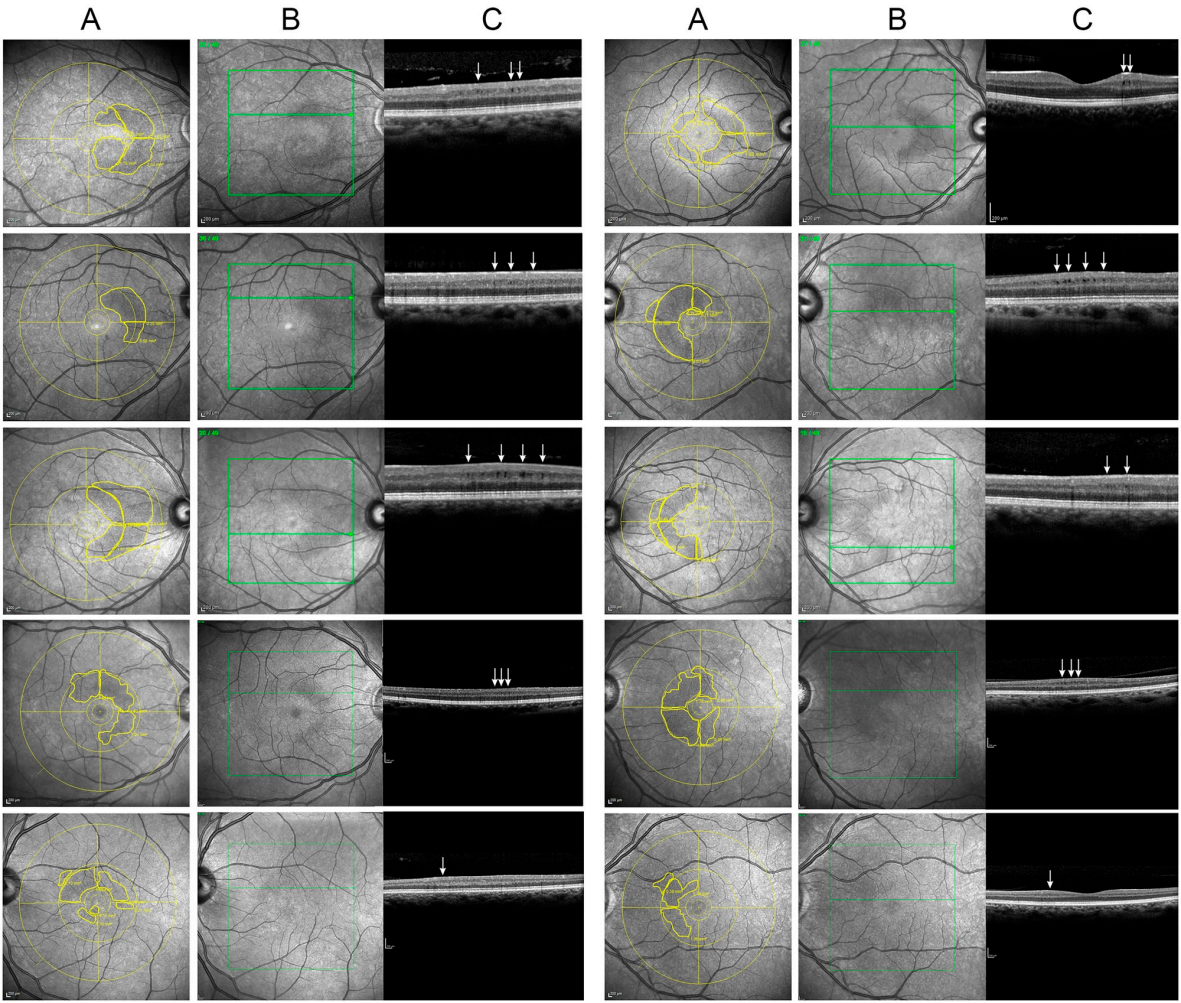
Heildelberg near infra-red reflectance images generated as separate images for the examiners (column A) and as reference images for optical coherence tomography line scans (columm B, not provided to the examiners) and representation of one of the 49 optical line scans submitted to critical evaluation of the presence of microcysts (arrows) in the inner nuclear layer (column C).

All subjects were also submitted to NIR imaging with the confocal scanning laser of the Heidelberg Engineering Spectralis equipment. The NIR imaging protocol involved the acquisition of images covering 30° of the posterior pole, using foveal fixation. Each NIR fundus image was analyzed for the presence of HA (scored as present or absent). The definition of HA was the occurrence of hyporeflectivity (as compared to the surrounding tissue), usually with a perimacular, most commonly semilunar shape and frequently with a dot-like pattern resembling a honeycomb, as reported elsewhere (Fig 1, columns A and B) [3,7]. Two reviewers masked to clinical status analyzed NIR images. The reviewers demarcated the outline of the HA areas observed in each macular quadrant using the area measurement software of the Spectralis equipment (Heidelberg Engineering version 1.7.1.0/HRA module 5.6.4.0) (Fig 1, column A). When the two reviewers disagreed regarding the presence or absence of HA, the NIR image was reviewed by a third examiner likewise masked to the clinical status of the eye. Outcomes agreed upon by two reviewers were considered as final. The total and hemi-macular HA areas measured by the two reviewers were then averaged, with the fovea as the central point.

### Statistics

The Kolmogorov-Smirnov test was used to verify the data distribution. The two groups were compared with regard to mean age using Student’s *t* test. OCT thickness parameters were compared using generalized estimated equation (GEE) models in order to account for inter-eye dependence when data from both eyes were used in comparisons. Pearson’s chi-squared test was used to evaluate categorical variables. Possible associations between the presence of HA and MMA were evaluated using Cohen’s kappa test. *P*-values of <0.05 were considered significant.

## Results

### Demographic data, VF and OCT-measured thickness

A total of 34 eyes with temporal hemianopia from 26 patients with previously treated chiasmal compression and 41 eyes from 22 healthy controls were studied. The fundoscopic examination revealed signs of BA of the optic disc and peripapillary RNFL loss in all 34 eyes. Twenty-four patients had diagnosis of pituitary adenoma, 1 craniopharyngioma and 1 suprasellar dysgerminoma. In 18 patients, only one eye met the inclusion criteria, while in 8 patients both eyes were analyzed. Table 1 displays information on age, sex, VF loss, and total and hemi-macular (nasal and temporal) average thickness for both patients and controls. All VF sensitivity parameters were significantly reduced in patients compared to controls. On the 24–2 VF test, 12 eyes had complete temporal hemianopia, 7 had a partial temporal VF defect greater than 1 quadrant, and 15 had a temporal VF defect of less than one quadrant. OCT-measured RNFL and GCL+ thickness was significantly smaller in BA eyes than in controls, while INL thickness was significantly greater (Table 1). Time-span between chiasmal decompression and the study ranged from 1 to 35 years. Mean ± SD age of subjects at the time of optic pathway decompression was 41.9 ± 10.5 years.

**Table 1:** Demographic variables in patients and controls along with visual field and optical coherence tomography findings in the eyes studied.

	Patients	Controls	P value
**Demographic**			
Subjects	26	22	
Eyes studied	34	41	
Age, yrs, mean (SD)	50.0 (10.7)	49.6 (6.3)	0.8^†^
Gender M/F	14/12	15/7	
**Visual field loss, mean (SD) in dB**			
MD	-8.4 (5.7)	0.2 (0.7)	<0.001*
TMD	-16.8 (11.4)	0.3 (0.8)	<0.001*
NMD	-1.8 (2.2)	0.1 (0.8)	<0.001*
**OCT thickness measurements (μm)**			
Total average RNFL	37.6 ± 4.0	46.8 ± 4.2	<0.001*
Total average GCL+	54.6 ± 11.1	76.4 ± 4.4	<0.001*
Total average INL	37.4 ± 5.0	30.4 ± 2.0	<0.001*
Nasal hemi-macula RNFL	40.7 ± 5.5	56.0 ± 5.2	<0.001*
Nasal hemi-macula GCL+	47.8 ± 10.9	76.0 ± 4.7	<0.001*
Nasal hemi-macula INL	40.9 ± 5.5	29.9 ± 2.5	<0.001*
Temporal hemi-macula RNFL	34.5 ± 3.2	37.6 ± 4.5	0.001*
Temporal hemi-macula GCL+	61.5 ± 12.3	76.7 ± 4.8	<0.001*
Temporal hemi-macula INL	33.9 ± 4.9	30.8 ± 2.1	0.001*

^†^ = Student’s *t* test;

* = generalized estimated equations models; SD = standard deviation; MD = mean deviation; dB = decibels; TMD = temporal mean deviation; NMD = nasal mean deviation.

### HA on NIR and MMA on OCT

HA was observed in 22 of 34 eyes with BA, but in none of the 41 controls (*p*<0.001, chi-squared test). Eyes with BA displayed HA more often in the nasal hemi-macula (n = 22) than in the temporal hemi-macula (n = 12) (*p* = 0.001). MMA was observed in 12 BA eyes and no controls (*p*<0.001, chi-squared test). Eyes with BA had MMA more often in the nasal hemi-macula (n = 12) than in the temporal hemi-macula (n = 6) (*p* = 0.001, chi-squared test). In BA eyes, HA (n = 24) was more prevalent than MMA (n = 12) (*p* = 0.03, chi-squared test).

In the 22 BA eyes with HA, the mean total HA area (± SD) was 5.3 ± 5.5 mm^2^. The corresponding figures for the nasal and temporal hemi-maculae were 3.9 ± 3.0 mm^2^ and 1.4 ± 3.1 mm^2^. In other words, the HA area was significantly greater in the nasal hemi-macula than in the temporal hemi-macula (*p*<0.001, GEE). In the 12 cases with MMA on OCT, the total mean number of microcysts (± SD) was 12.8 ± 10.2. The corresponding figures for the nasal and temporal hemi-maculae were 11.0 ± 8.0 and 1.8 ± 2.5. Thus, the number of microcysts was significantly greater in the nasal hemi-macula than in the temporal hemi-macula (*p*<0.001, GEE).

### Agreement between examiners

The agreement between the two examiners was estimated with regard to the presence/absence of HA on NIR and MMA on OCT. As for HA, the examiners concurred in their assessment of all control eyes and 31 of 34 eyes with BA, indicating a very high level of agreement (k = 0.88, *p*<0.001). As for MMA (in any location of the macula), the examiners concurred in their assessment of all 75 eyes examined, although the microcyst count varied significantly.

### Agreement between HA on NIR and MMA on OCT

Table 2 shows the presence/absence of HA and MMA in all 34 eyes with BA of the optic nerve and the level of agreement between the two parameters using Cohen’s kappa coefficient: 0.24 (total), 0.29 (nasal hemi-macula) and 0.27 (temporal hemi-macula).

**Table 2:** Number of eyes with microcystic macular abnormality (MMA) and hyporeflective abnormality (HA) on confocal infrared photography in 34 eyes with temporal hemianopia and band atrophy of the optic nerve.

	HA+	MMA+	HA+ MMA+	HA- MMA-	HA+ MMA-	HA- MMA+	Kappa*
**Total average**	22	12	10	10	12	2	0.24
**Nasal hemi-macula**	21	12	10	11	11	2	0.29
**Temporal hemi-macula**	12	6	4	20	8	2	0.27

* = Cohen’s kappa test; MMA = microcystic macular abnormality; HA = hyporeflective abnormalities; NHM = nasal hemi-macula, THM = temporal hemi-macula. The + and–signs indicate the presence and absence of abnormality, respectively.

### OCT and VF findings: HA+MMA+ versus HA-MMA-

**Fig 2:**
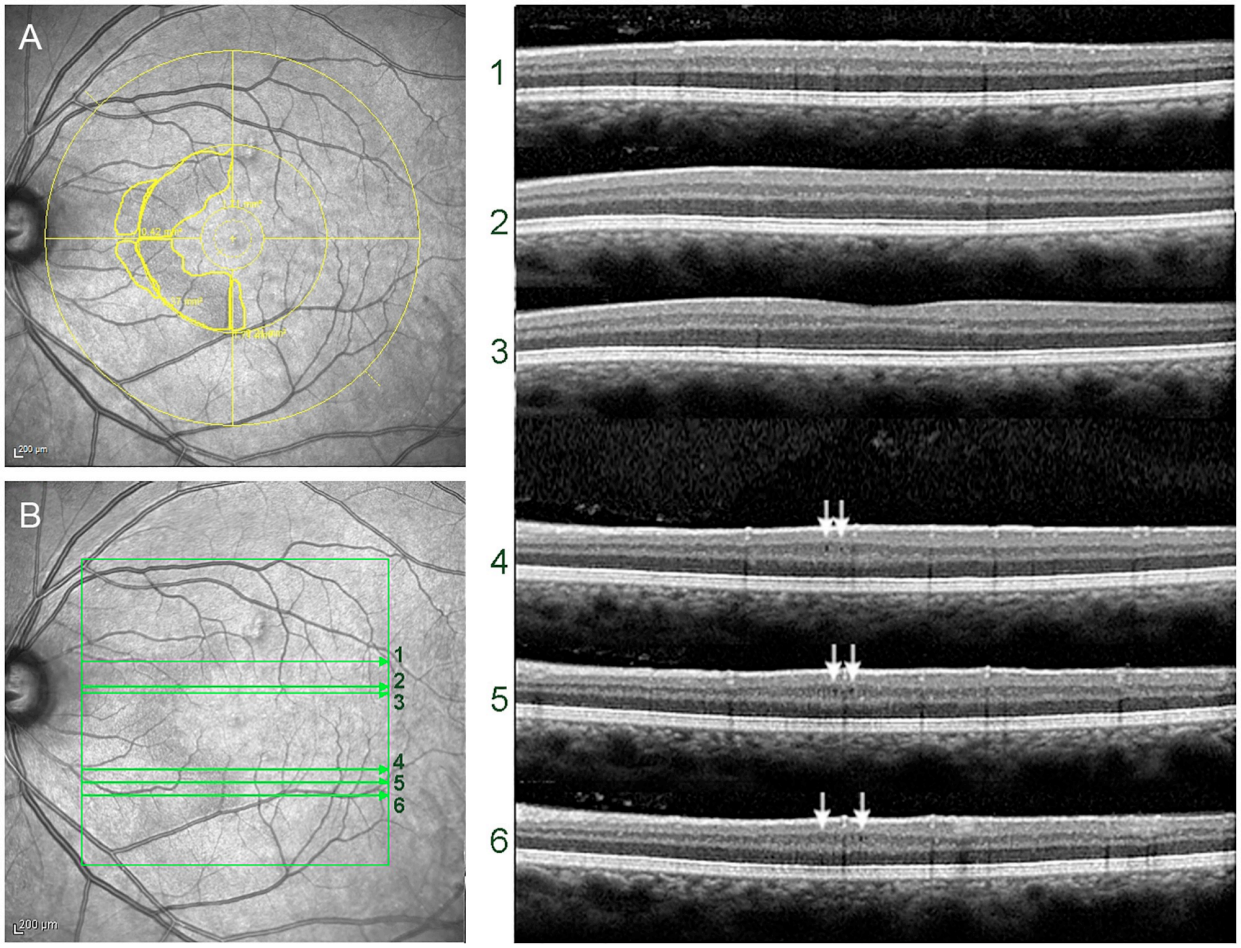
A: Example of NIR reflectance image showing hyporeflective abnormalities (HA) demarcated in a semilunar pattern in the nasal hemi-macula. B: The same picture as A, with B-scan lines (numbered 1 to 6) corresponding to those on the right. Note that OCT scan lines 1–3 display no microcysts despite coinciding with the HA area, while lines 4–6 are a positive match between HA and MMA (white arrows).

Fig 1 depicts 10 eyes with both HA on NIR and MMA on OCT (HA+MMA+). Although both findings were present, MMA could not be detected on OCT along the entire HA area outline demarcated on NIR. Fig 2 shows an example of NIR and OCT findings for an eye labeled as HA+MMA+. HA was drawn in a semilunar area nasally to the fovea, directly corresponding to moderate temporal VF defects limited to the temporal field (TMD = -6.95 dB). On the other hand, MMA was only detected in some line scans on similarly-looking NIR images, indicating that the OCT technology employed in this study was unable to detect MMA in several areas with clear evidence of RMM on NIR.

In 10 eyes, neither NIR nor OCT detected any abnormality (HA-MMA-), despite severe axonal and VF loss. To explore this point further, we compared 10 HA+MMA+ eyes to 10 HA-MMA- eyes with regard to macular thickness and VF. Five HA+MMA+ eyes had complete temporal hemianopia and 5 displayed a temporal VF defect greater than one quadrant. Three HA-MMA- eyes had complete temporal hemianopia and 7 displayed a VF defect of less than one quadrant (Table 3). In all comparisons, the INL was significantly thicker and VF loss was more severe (though not significantly) in HA+MMA+ than in HA-MMA-. No significant difference was found regarding total or hemi-macular RNFL and GCL+ measurements.

**Table 3:** Comparison of visual field and inner retinal layer thickness measurements in two subsets of eyes with temporal hemianopia and band atrophy of the optic nerve, with or without hiporeflective abnormality on near infrared photography and microcystic macular abnormality on OCT (HA+MMA+ and HA-MMA-, respectively).

	HA+MMA+ (n = 10)	HA-MMA- (n = 10)	P value*
Visual field loss, mean (SD) in dB			
MD	-11.2 (4.3)	-6.9 (5.6)	0.06
TMD	-22.7(8.4)	-14.7 (12.2)	0.09
NMD	-2.1 (2.1)	-0.8 (1.2)	0.08
Total average OCT measurements (μm)			
RNFL	37.3 ± 2.4	36.9 ± 2.7	0.74
GCL+	53.1 ± 17.0	54.1 ± 7.5	0.86
INL	41.6 ± 5.0	35.3 ± 4.2	*0*.*001*
Nasal hemi-macula (μm)			
RNFL	40.0 ± 3.3	39.9 ± 4.1	0.94
GCL+	47.6 ± 16.4	46.5 ± 6.6	0.84
INL	45.5 ± 6.0	38.9 ± 4.8	*0*.*004*
Temporal hemi-macula (μm)			
RNFL	34.6 ± 2.7	33.9 ± 1.6	0.52
GCL+	58.6 ± 18.1	61.8 ± 9.3	0.63
INL	37.7 ± 4.6	31.7 ± 3.7	*0*.*001*

* = generalized estimated equations models (GEE); SD = standard deviation; MD = mean deviation; dB = decibels; TMD = temporal mean deviation; NMD = nasal mean deviation.

### Critical analysis of disagreement between HA on NIR and MMA on OCT

**Fig 3:**
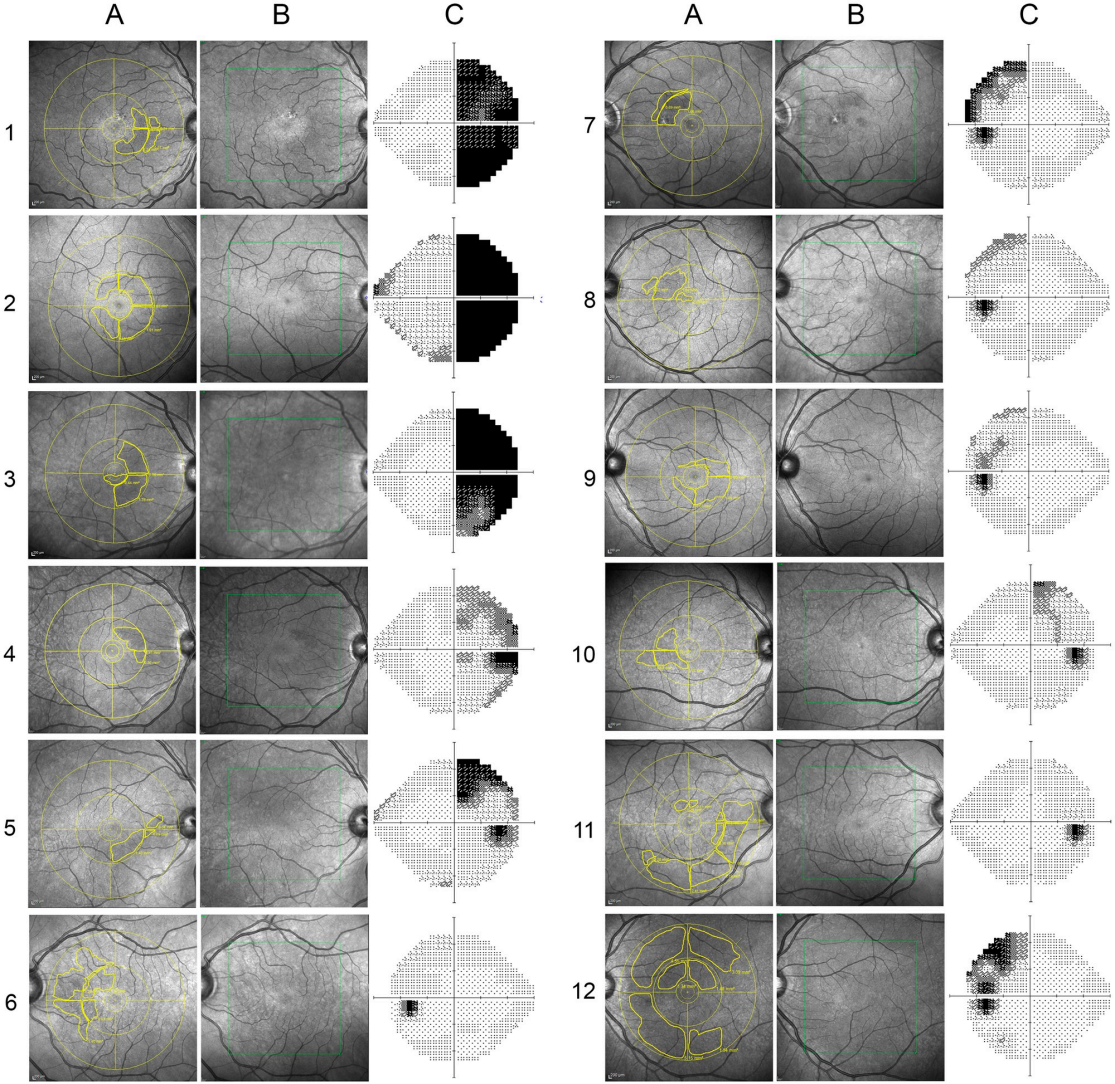
Near infra-red reflectance images and visual fields of 12 eyes labeled as having hyporeflective abnormalities but no microcysts on OCT (HA+MMA-). In eyes 1–8, the shape and location of the HA area (nasal hemi-macula, corresponding to the temporal VF defect) allowed to label it a true positive. In eyes 9–12, the location and/or size of the demarcated HA area, when compared to the extension of the VF defect, allowed to label it a false-positive.

**Fig 4:**
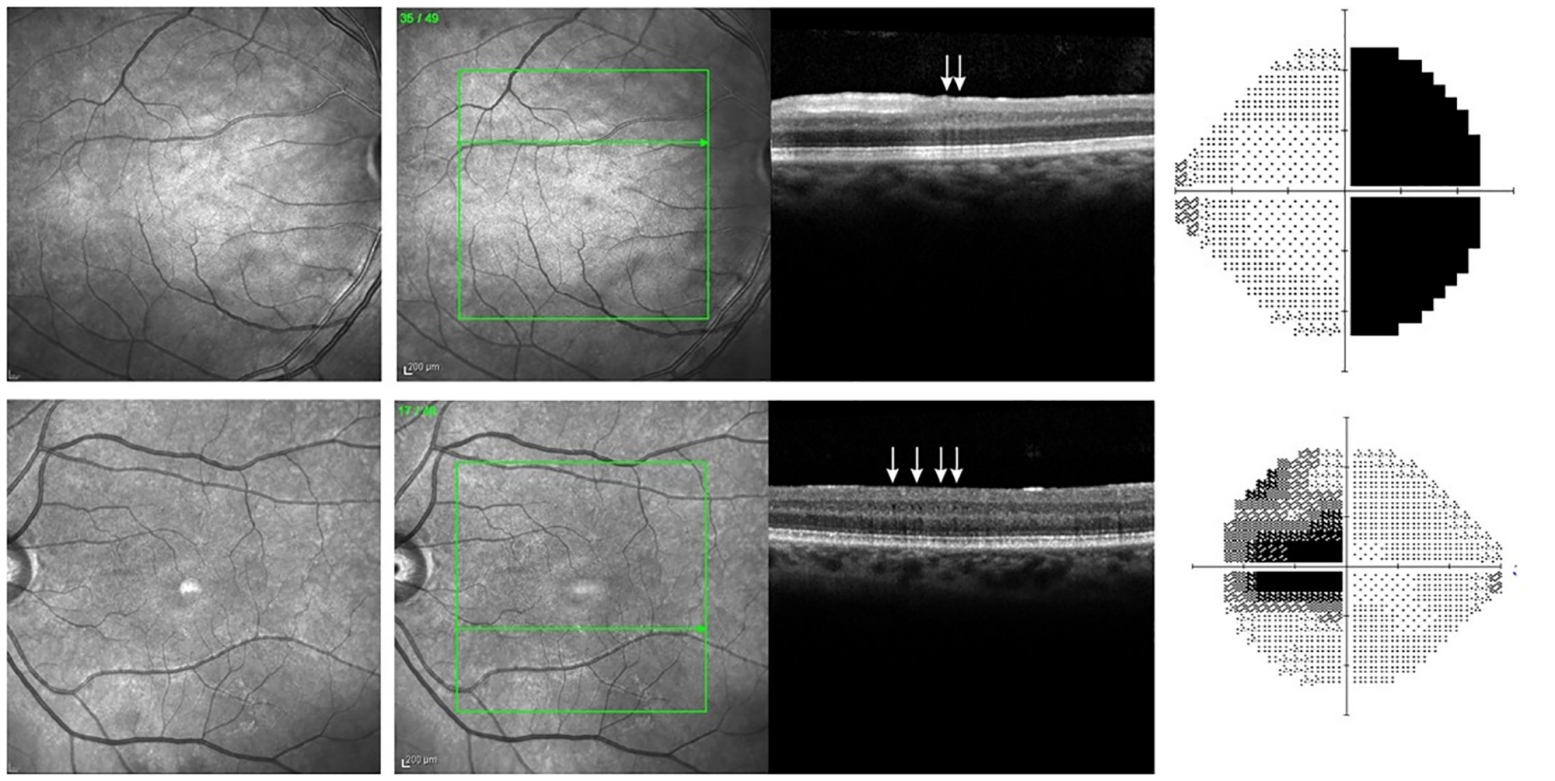
Near infrared reflectance imaging (two first columns) of 2 patients labeled as having no hyporeflective abnormalities (HA-MMA+) by the examiners. Optical coherence tomography scans showing microcysts in the inner nuclear layer (white arrows, third column). The two patients had dense temporal VF defects (right column).

Fourteen eyes displayed either HA on NIR or MMA on OCT, but not both. To explore this discrepancy, a reviewer aware of the results of the blinded examiners critically evaluated each case, including the corresponding VF defect. Fig 3 shows the NIR and VF findings of 12 eyes labeled as HA+MMA-. In 8 eyes (1–8, Fig 3), a careful analysis of the location and demarcated outline of the HA area (normally expected to be predominant in the nasal hemi-macula corresponding to the temporal VF defect) led to the conclusion that HA was most likely not a false-positive finding. In the remaining 4 eyes (9–12, Fig 3), the diagnosis of HA was most likely false-positive, either because HA was restricted to the temporal hemi-macula or because the demarcated area was too large to correspond to the respective VF loss. Fig 4 shows the only 2 eyes labeled as HA-MMA+ (with MMA detected in at least two sequential scans). Both displayed severe temporal VF loss. Based on our analysis of the eyes in Figs 1, 3 and 4, we believe RMM was correctly diagnosed in 20 eyes. OCT detected MMA in 12 eyes indicating a sensitivity of 60%. Since the abnormality was defined based on OCT, there was no eye in which the equipment was though to overcall the finding. On the other hand, NIR correctly detected RMM in 18 out of 20 eyes, overcalled 4 and missed 2, leading to an estimated sensitivity of 90% and a specificity of 71.4%.

## Discussion

The current study shows that RMM occurs in a large percentage of patients with long-standing compressive chiasm lesion. Currently believed to indicate RMM, MAA was originally defined based on OCT findings of cystic, lacunar areas of hyporeflectivity in INL in patients with inflammatory optic neuropathies [4,23]. The same criteria were used in subsequent studies evaluating different types of optic neuropathies [2,3,5–7,14,24–26].

In the present masked analysis, HA was diagnosed on NIR in 22 of 34 eyes with temporal hemianopia and BA, while MMA was observed in 12 eyes. Neither abnormality was found in any control eye. In a post- analysis taking into consideration OCT, NIR and VF findings, we concluded that some degree of RMM was present in 20 of the 34 eyes (58.8%). This high prevalence, if compared to other optic nerve diseases, may in part be explained by the severity of optic nerve damage in the population from which our sample was drawn. As shown by earlier studies, RMM is strongly correlated with visual loss severity [4,7,27]. In a study on optic neuritis, MMA was found in 6.3% of eyes of patients with multiple sclerosis, 21% with neuromyelitis optica spectrum disorder (NMOSD) and in 53.8% of eyes with chronic relapsing inflammatory optic neuropathy (the latter two associated with much worse visual outcome than multiple sclerosis) [7]. In addition, the occurrence of MMA appears to be more common in young patients [2,27], especially in children and young adults with degenerative optic nerve disease, but rather uncommon in older glaucoma patients. In fact, according to one hypothesis, the incidence of MMA depends on the type of optic neuropathy. Thus, Kessel and coworkers [27] reviewed the literature and concluded that MMA is present in 65.4% of eyes with hereditary optic neuropathy and 14.3% of eyes with NMOSD, but relatively uncommon in patients with glaucoma and multiple sclerosis (<4.0%). In the only previous study evaluating the occurrence of MMA in a large series of eyes with compressive optic neuropathy, we retrospectively analyzed OCT scans of eyes with chiasmal compression and found MMA in 21.2% [5]. However, the resolution of the OCT device used in that study was lower than that of the current study, and no analysis of NIR images was performed. The increase in OCT resolution and the careful analysis of individual line scans by two examiners may explain the high incidence of RMM detected on OCT as MMA (35.3%) compared to the previous study. The combination of NIR and OCT analysis raised the detection of RMM to 58.8%.

Our results are supported by several studies reporting an association between RMM and increased INL thickness and decreased macular RNFL and GCL thickness [2,5,7] (Tables 1 and 2). Therefore, although initially attributed to retinal inflammation, RMM appears to be directly correlated with retinal axonal loss expressed as RNFL and GCL thinning. However, there is still no consensus as to the pathophysiological mechanism of RMM development. Some authors believe vitreous traction plays a role in the emergence of MMA [6,28], but this seems unlikely primarily because MMA was clearly predominant in the nasal hemi-macula directly corresponding to the VF defect. Also, in many eyes RMM exclusively affected the nasal hemi-macula (Fig 2), matching the findings of a previous study [5]. Since it would be difficult to account for a tractional effect respecting the vertical meridian in the macula, we are inclined to dismiss the vitreous traction hypothesis. Some have pointed out the involvement (or dysfunction) of Müller cells in the development of MMA [8,29]. Müller cells traverse the retina from the inner limiting membrane near the vitreous to the external limiting membrane and play an important role in the maintenance of the retinal framework. RNFL and RGC atrophy could therefore exert a tractional effect on Müller cells, resulting in compensatory INL thickening and eventually leading to the formation of vacuolar areas of microcysts. Findings from a previous study using multifocal electroretinography and OCT in eyes with BA of the optic nerve suggest that INL thickening and MMA may result from the combination of mechanical stretching of the retinal structures and secondary degeneration of bipolar, amacrine and Müller cells in the INL [8]. Müller cells could conceivably act as a scaffold holding the architecture of the retina, despite advanced ganglion and RNFL loss, therefore stretching the inner nuclear layer. Younger subjects with relatively rapid progression of visual loss would be more prone to this phenomenon, especially if, as believed, Müller cells remain largely unaffected by degeneration. Following the same line of reasoning, older individuals with chronic disease, slow progression of visual loss and progressive Müller cell collapse would be less likely to develop MMA. Speculative as they may be, the above considerations are intended to broaden the scope of investigation into the causative mechanism of MMA.

In this study, we not only determined the incidence of RMM in a sample of patients with temporal hemianopia from chiasmal compression, but, more importantly, compared the ability of NIR and OCT to detect RMM. Other authors have employed NIR to detect HA [2,3,30], but the use of HA to diagnose RMM, without visualizing MMA on OCT, has not been attempted before. In a retrospective analysis of patients with demyelinating diseases, Kaufhold et al. [30] found MMA on OCT and HA on NIR in 22 eyes of 283 patients. An independent examiner blinded to the OCT findings who subsequently evaluated the NIR images of all the patients confirmed the diagnosis of HA in the 22 eyes above, but found abnormalities in another 27 eyes. Considering these cases to be false-positives, the authors concluded NIR had a sensitivity of 100% and a specificity of 95.2%. On the other hand, if these positives were real, the sensitivity of OCT to detect deeper retinal layer abnormalities in single retinal scans would not be as high as that of NIR. The authors did point out that 3 of the 27 eyes with abnormal NIR images were suspected of MMA but did not meet the criteria for visible abnormalities on two adjacent OCT scans [30].

To evaluate the diagnostic ability of NIR and OCT to detect RMM, two experienced examiners masked to the clinical status of the eye evaluated the NIR images and OCT scans of all 75 eyes. Blinded to the NIR data and working separately, the examiners carefully searched all 49 scans for microcysts in the INL. Our findings suggest that, in BA eyes, HA was significantly more frequent than MMA (*p* = 0.03), and that, in many HA+MMA+ eyes, microcysts were not evident in all line scans coinciding with the HA area (Fig 2). This clearly indicates NIR is more sensitive than OCT at detecting RMM. One might at first be tempted to suspect the difference is due to false-positives; after all, since RMM was originally defined based on OCT findings of MMA, all positives on OCT are necessarily true, whereas an alternative method of detection like NIR might be more prone to false-positive results. However, a careful analysis of our cases shows that, although 4 cases of HA on NIR were very likely false-positive, the sensitivity of NIR is higher than that of OCT, even when the resolution is high and 49 individual line scans are analyzed. On the other hand, in two eyes microcysts were visible in some line scans despite the fact that two blinded observers agreed on the absence of HA on NIR. Some such cases might be explained by light exposure, unique fundus pigmentation or examiner error (Fig 4), but the possibility of NIR missing some cases of RMM can not be disregarded and, ideally, the two methods should be used in combination for a more reliable diagnosis.

One explanation for the relatively low sensitivity of OCT detection of MMA (as compared to HA on NIR) to a high incidence of false-negatives by the current examiners, but this seems unlikely since both searched specifically and thoroughly for MMA in all 49 B-scans and a perfect agreement between them was observed when adhering strictly to the criterion of microcysts been present in two consecutive scans (despite some variation in the number) [4]. We believe the following explanations are more plausible: i) by averaging 16 high-resolution scans in each line in order to improve quality (and despite the use of the software TruTrack), MMA may have gradually disappeared when averaging the results of several scans, making it harder to detect small cysts. In other words, using a smaller average number of line scans might improve MMA detection despite possible loss of definition; ii) the presence of MMA may depend on the retinal degeneration stage, making it more evident in young eyes with inner retinal layer loss but relative preservation of other types of cells, especially Müller cells. On the other hand, in older individuals with chronic disease and slow progression, MMA might shrink due to the progressive collapse of Müller cells, making MMA detection more difficult while still allowing for HA to be visualized on NIR; and iii) INL thickening secondary to axonal loss may need to reach a certain level to make MMA formation possible, but may be enough to reveal HA on NIR (which does not require cystic areas to be detectable). Further studies on NIR, if possible combined with high-resolution OCT technology and alternative averaging protocols, are necessary to fully understand the issue.

In conclusion, our study indicates that RMM is more prevalent than previously thought in eyes with severe visual field loss from chiasmal compression and that it may be more easily detected on NIR than on individual OCT line scan evaluation. Therefore, when searching for the presence of RMM, NIR may be useful both in the conjunction with OCT as well as a screening method, to suggest careful perusal of OCT scans to confirm its finding.

## Supporting information

S1 File Microcystic maculopaty study raw data. (XLSX)
